# Supplementary material for: A human pathogenic hantavirus circulates and is shed in taxonomically diverse rodent reservoirs
Source: PLoS Pathog. 2025 Jan 21;21(1):e1012849. doi: 10.1371/journal.ppat.1012849 (PMC11785344; doi:10.1371/journal.ppat.1012849)
Supplement: S1 Fig — (A) Experimental design using tissue homogenates of heart, kidney and/or lung from screened SNV-positive rodents are used to deliver virus to PMVEC from deer mice lung tissue and detected through RT-qPCR. (B) Copies per mL of SNV for different tissue homogenates during infection at multiple time points. No template control (NTC) and positive (Pos) control were used for threshold adjustment. Images created with Biorender.com. (DOCX) [file ppat.1012849.s001.docx]

**
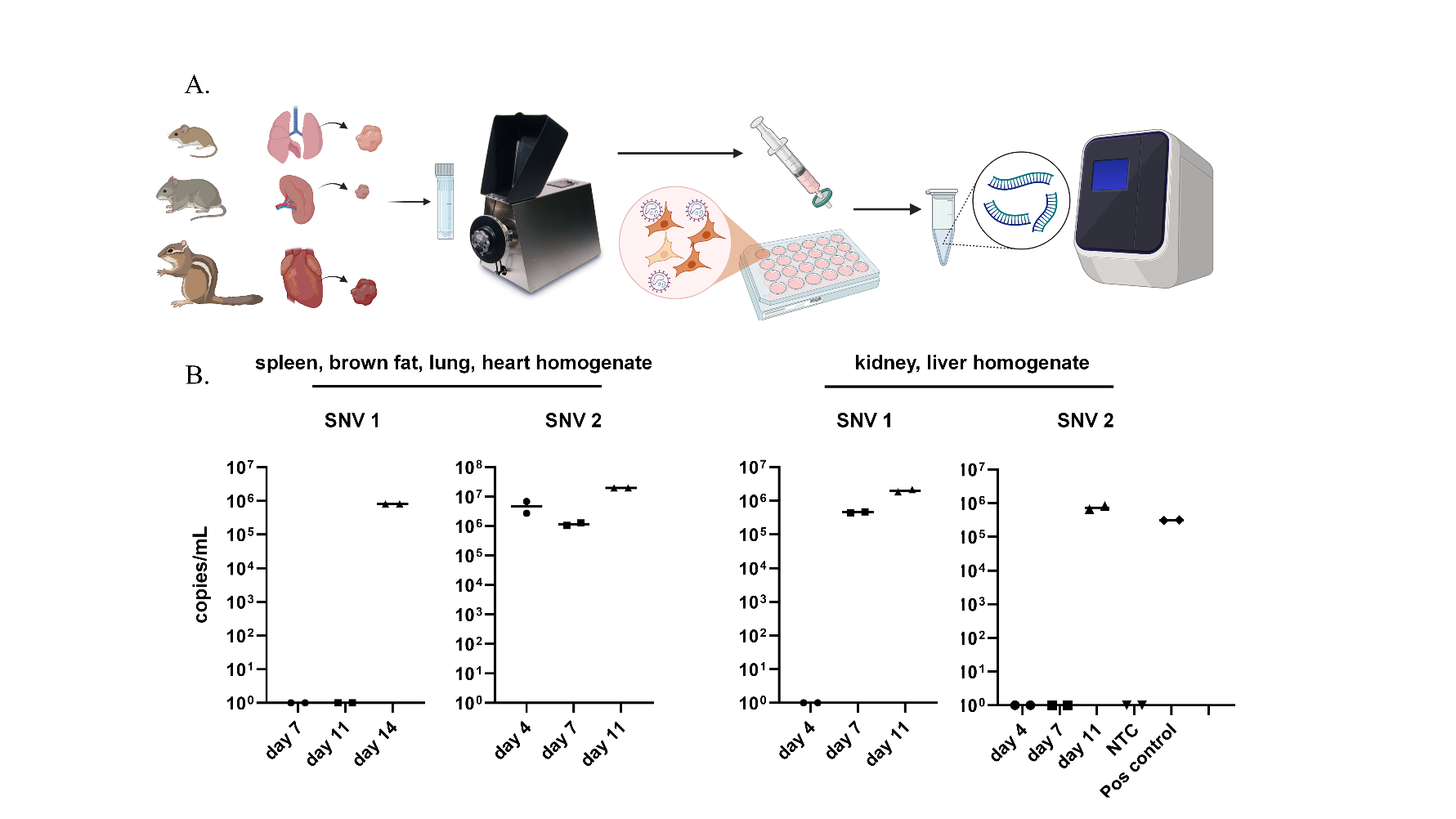
Supplemental Figure 1. Pulmonary microvascular endothelial cells (PMVEC) deer mouse derived cells can be infected by SNV-positive tissue homogenates.** (A) Experimental design using tissue homogenates of heart, kidney and/or lung from screened SNV-positive rodents are used to deliver virus to PMVEC from deer mice lung tissue and detected through RT-qPCR. (B) Copies per mL of SNV for different tissue homogenates during infection at multiple time points. No template control (NTC) and positive (Pos) control were used for threshold adjustment.
