# Supplementary material for: A human pathogenic hantavirus circulates and is shed in taxonomically diverse rodent reservoirs
Source: PLoS Pathog. 2025 Jan 21;21(1):e1012849. doi: 10.1371/journal.ppat.1012849 (PMC11785344; doi:10.1371/journal.ppat.1012849)
Supplement: Attachment — Submitted filename: SNV_HostReservoir_PP_ReviewersResponse_final_12_6.pdf [file ppat.1012849.s002.pdf]

We would like to thank the reviewers for taking the time to provide a rapid, thorough review with helpful comments and suggestions regarding our work. Below we have addressed each point in *italics*.

Reviewer's Responses to Questions

Part I - Summary

Please use this section to discuss strengths/weaknesses of study, novelty/significance, general execution and scholarship.

Reviewer #1: The report by Goodfellow et al. provides important data to support an expanded view of the species involved in maintenance and transmission of hantaviruses. While evidence of hantavirus infection (predominantly by serology) has indicated that other host species are susceptible to infection, it was believed that maintenance and transmission of each hantavirus species was linked to one main host. This report has many strengths including, but not limited to the large sample set representing various years of sampling; including more recent samples and archive samples; a wide diversity of putative host species tested; evaluation of both tissues and other biological samples (feces and salivary glands); PCR analyses and infectious virus isolation; and analysis of data with consideration for epidemiology of human cases. The advances provided in this report are key to expanding knowledge of hantavirus ecology. The only weaknesses in this report are areas that could be revisited for clarity and readability which are described below in “Part III – Minor Issues” section of review. Importantly, this report would be strengthened by expanding table and figure legends with more details of the samples tested, sample sizes and making sure it is very clear which data represent RNA quantification alone vs. infectious virus.

Reviewer #2: This is a very large survey of Sin Nombre virus (SNV) in wild-caught small mammals in New Mexico. Sin Nombre is a bunyavirus in the genus orthohantavirus, that can cause fatal disease. The primary reservoir for SNV is the western deer mouse that is widely distributed throughout the United States. In New Mexico, the vast majority of the human cases occur in the northwestern part of the state. However, seroprevalence studies suggest SNV is present elsewhere in the state. Previous studies also suggested that other small mammal populations are positive for SNV. The authors found that 25% of the nearly 1600 lung samples were positive for SNV RNA and that many different mammal species were positive. Next they attempted to isolate the virus from 90 different samples from different species. After several passages, SNV RNA was detected in many different tissues and species.

This manuscript is an follow up on a paper published earlier this year in PLoS One by the same group describing the Distribution and prevalence of Sin Nombre hantavirus in rodent species in eastern New Mexico. In this study they tested for SNV RNA in over 700 specimens and concluded that SNV RNA could be detected in many different small mammals across the state, reducing some of the novelty of the work presented here. Aside from that, no virus sequence data is presented. Comparisons of sequences between different mammal species and locations in New Mexico may offer insights into why most of the human cases of SNV occur in the northwestern parts despite the presence of the virus in other parts.

Part II – Major Issues: Key Experiments Required for Acceptance
Please use this section to detail the key new experiments or modifications of existing experiments that should be absolutely required to validate study conclusions.

Generally, there should be no more than 3 such required experiments or major modifications for a "Major Revision" recommendation. If more than 3 experiments are necessary to validate the study conclusions, then you are encouraged to recommend "Reject".

Reviewer #1: No major issues.

Reviewer #2: Gene sequence data needs to be included to (a) confirm that these are unique viruses and isolates that were expanded, (b) to add significance to the paper, and (c) identify species or location differences in the SNV genome sequence. Even just sequencing the envelope protein on all the cultured viruses and some of the original tissues with high virus loads would greatly improve this paper.

We agree that this is a very important topic; in fact, we have a separate manuscript in preparation where we are analyzing dozens of sequences directly from the lungs of animals (as well as human patient samples) to address this question. Hantaviruses in hosts are extremely difficult to sequence. We have spent >3 years conducting PCR tiling and subsequent NGS sequencing, followed by Sanger sequencing of PCR fragments to fill in gaps, to get acceptable coverage of these viruses from tissues. We are hoping to compile this data in the coming months for phylogeny analysis; this would comprise the largest set of genomes for Sin Nombre virus ever reported. We feel that due to the rarity of these data, the years worth of work it took to get the data, and the large amount of data in the current manuscript, it is reasonable to publish the sequencing data separately. However, we appreciate and understand this comment, so below is a preliminary phylogeny of the M segment coding region for the glycoproteins of SNV. This data and additional sequence information for L and S will be included in a separate manuscript to be submitted soon. We have also added text to the discussion that covers the importance of getting this sequencing data and stating that this is the next step to understanding Sin Nombre virus epidemiology. We feel that the isolation is significant and novel enough to stand on its own for emphasizing the reservoir and prevalence distribution.

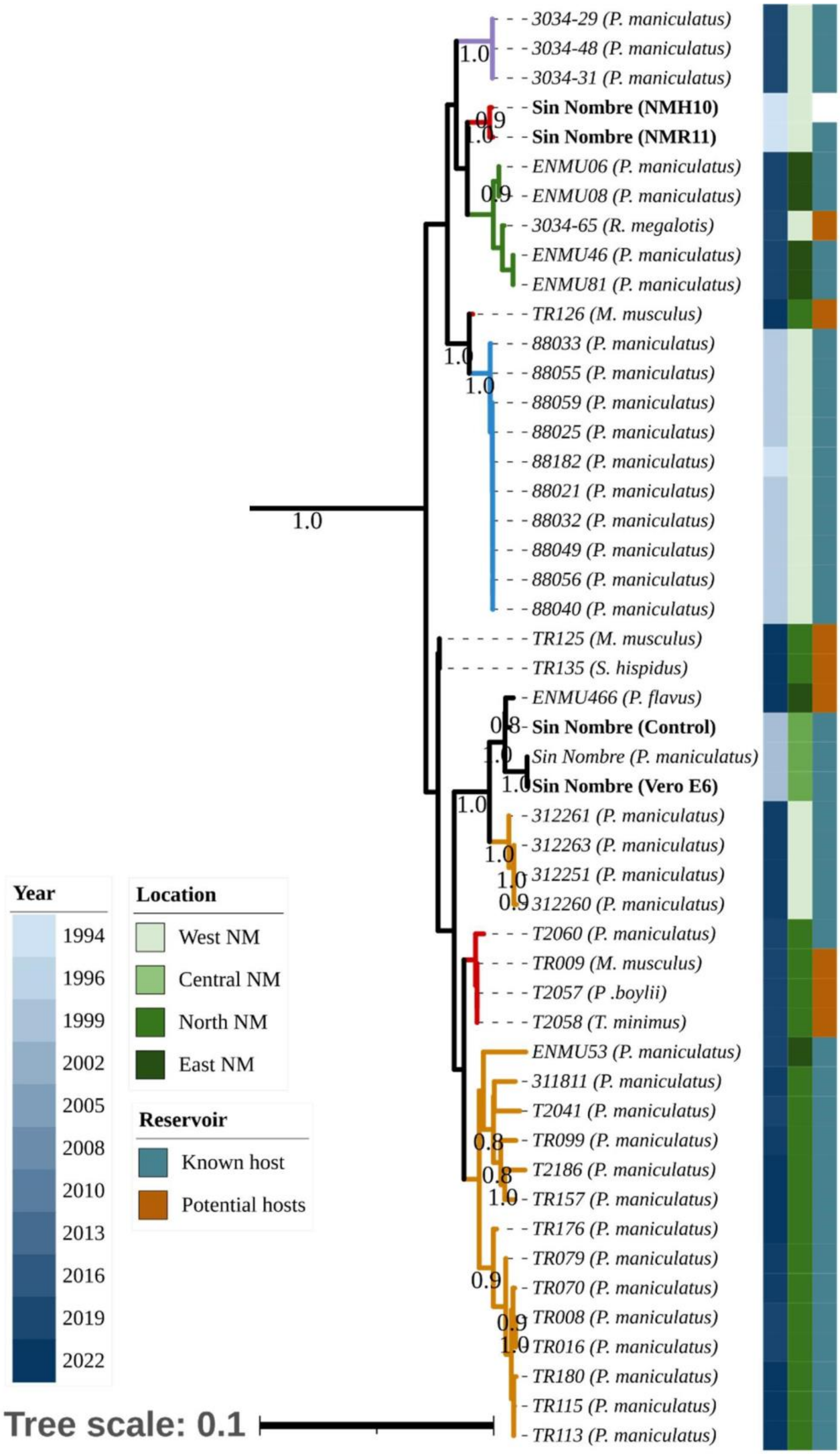

Figure. M segment sequences of SNV from various wild-caught small mammals throughout New Mexico between 1994-2022.

Can the authors explain how the virus in Figure 4 can go up and down for some of the isolates? And how does this compare to a control virus?

The virus itself increases due to the higher viral load detected during the different days of infection. However, the drastic decrease followed by an increase in the next passage, which we found in very few of our samples, may be attributed to an error in collection and extraction for detectable nucleic acids for those particular samples. However, we wished to include these data for transparency.

Part III – Minor Issues: Editorial and Data Presentation Modifications
Please use this section for editorial suggestions as well as relatively minor modifications of existing data that would enhance clarity.

Reviewer #1: Line 27: Please clarify what "25% frequency rate" refers to. Overall (all years/species sampled)? Average of mean at individual sites? The study is quite extensive and it's not clear in abstract what the value encompasses.

This has been clarified and reflected in lines 27 and 28 of the track changes document. We have also gone over the data thoroughly and found minor changes to the SNV positivity rate, which increased to 26.6%

Line 49: Can you please specify what the case count is to date in NM (vs. just saying "highest").

This information has been added to lines 67 and 68.

Line 77: While sampling is detailed in M&M, it would be helpful to list briefly here also. For samples from 2019-2023 especially, as lung alone is specified for the archive samples. It would be helpful to specify that terminal sampling was performed.

This information has been added between lines 96-99.

Line 77-88: Please specify, if possible, if there were months/seasons where sampling was performed, or year-round?

The seasonal range was added in lines 96-97.

Table 1: Please add a column with common names in addition to species. Common names are mentioned

later in the paper, and it would be helpful to have them in this table. Also please clarify if the "# screened (% total)" include the archive or is independent. If independent and represents the newer samples, is it positive on one or more tissues? Lung alone? Would be helpful to clarify which tissues were considered for other column given lung is specified for last column.

A column for common names has been added. Additionally, some language has been included in the figure caption to specify that museum archive tissues were also included in the table. We removed "lung tissue" and renamed as "tissue" to avoid any confusion while re-emphasizing that a majority of tissues tested were lung, however liver and kidney were tested for some specimens when lung was not available (51 samples, or about 3.3%). These corrections are within the figure caption and manuscript text.

Line 161: Does "SNV lung titer" refer to RNA or infectious virus? Not clear as written. Because assays are done to look at vRNA and others with intent to assess infectious virus, please review text and specify vRNA load/titer when infectious virus not assessed. It is confusing in some sections which data reflects infectious virus vs. RNA alone and this is a key aspect (and important advancement) provided in this work.

Thank you for this comment and we agree, this has been clarified throughout the manuscript.

Figure 3: The authors do a great job of linking data to geography, but it would be helpful to have more transparency about sample sizes, as they vary (for example in 3D). Please add details of sample sizes to text and figure legends wherever possible in this figure, and throughout, to aid readers in interpretation of data and to increase utility for comparison with future studies.

We agree and have changed Figure 3 to graphically display the sample size at each trapping site.

Table 2: Please clarify the sampling dates in table legend, in addition to that noted in text. Also clarify if only vRNA assessed and if only in Lung (as opposed to table 1 which may include other tissues?). Location # and sites are listed. Does the report include a key or more info regarding where in the state these locations are? Include site number info in Figure 3 or in supplemental table?

Clarification of the information requested has been added to Table 2's figure legend. A reference to our previous publication of this case study has been included which contains additional information regarding the trap sites.

Line 232-238: Developed isolation protocol will be of great use for the field. However, there is no section in M&M that clearly outline isolation protocol (cell culture mentions lung endothelial cells – should use PMVEC acronym here to be clear?) – also not clear what antibiotics/antifungals were added when attempting isolation from different sample types (specify if none used because this is important point).

Thank you for this comment; we have added PMVEC for clarification while also specifying the media used. We also added our description of culturing conditions (for example, fecal samples were sterile

filtered). We have also grouped the conditions for SNV isolation from tissues under a new heading entitled "Live SNV isolation from tissue samples" to clarify our approach.

Line 239: How were the 90 samples used to attempt virus isolation selected? Based on Ct values? Cut-off? representative of different species? Please clarify.

Clarification has been added to lines 218-219.

Reviewer #2: Review Fig S1. The arrow suggests cells were put into animals?

This has been corrected to more adequately depict the procedure.

This reviewer also suggests to put the high SNV RNA positivity rate into context. For example, is SNV causing a crhonic or an acute infection in mice and other rodents? If acute, how do you explain this high rate of SNV positivity? Are the infected animals more likely to get caught? Or does it cause a chronic life long infection?

We appreciate the questions and agree that they are interesting. According to Hjelle and Yates 2001, hantaviruses in rodent hosts are chronic infections with little to no pathologic effects or signs of disease. There is little to no data suggesting infected animals get caught more frequently, however during infestations at sites of human infection, a higher chance of capturing positive infected animals can occur but is variable. For example, in Utah, 29.7% antibody prevalence in the rodent populations as a consequence of human disturbance was reported (Mackelprang et al. 2001). Another long-term study was performed at Yosemite where seroprevalence varied based on year or location between 4.8 to 14.5% (Danforth et al. 2012). Lastly, an earlier study was conducted in national parks in regions that had no reported human SNV cases that showed antibody prevalence in 7% of deer mice populations, but also was dependent on location, where northeast regions had the highest prevalence (Mills et al. 1998). Altogether, the variability between studies appears to be dependent on location, rodent populations and species diversity, along with frequency of human spillover. We have added a section to the discussion to address this point.

Hjelle B. and Yates T.. 2001. Hantaviruses. Modeling Hantavirus Maintenance and Transmission in Rodent Communities. C. S. Schmaljohn and S. T. Nichol (eds.). Springer-Verlag., Berlin, Germany, pp. 77–90.

Danforth, M. E., Messenger, S., Buttke, D., Weinburke, M., Carroll, G., Hacker, G., Niemela, M., Andrews, E. S., Jackson, B. T., Kramer, V., & Novak, M. (2020). Long-Term Rodent Surveillance after Outbreak of Hantavirus Infection, Yosemite National Park, California, USA, 2012. Emerging infectious diseases, 26(3), 560–567. https://doi.org/10.3201/eid2603.191307

Mills, J. N., Johnson, J. M., Ksiazek, T. G., Ellis, B. A., Rollin, P. E., Yates, T. L., Mann, M. O., Johnson, M. R., Campbell, M. L., Miyashiro, J., Patrick, M., Zyzak, M., Lavender, D., Novak, M. G., Schmidt, K., Peters, C. J., & Childs, J. E. (1998). A survey of hantavirus antibody in small-mammal populations in selected United States National Parks. The American journal of tropical medicine and hygiene, 58(4), 525–532. https://doi.org/10.4269/ajtmh.1998.58.52

PLOS authors have the option to publish the peer review history of their article (wha t does this mean?). If published, this will include your full peer review and any attached files.

If you choose “no”, your identity will remain anonymous but your review may still be made public.

Do you want your identity to be public for this peer review? For information about this choice, including consent withdrawal, please see our Privacy Policy.

Reviewer #1: No

Reviewer #2: No
